# Sama: a contig assembler with correctness guarantee

**DOI:** 10.1186/s13015-025-00280-y

**Published:** 2025-06-03

**Authors:** Leena Salmela

**Affiliations:** Department of Computer Science, University of Helsinki, Pietari Kalmin katu 5, FIN-00014 Helsinki, Finland

**Keywords:** Genome assembly, De Bruijn graph, Correctness guarantees

## Abstract

**Background:**

In genome assembly the task is to reconstruct a genome based on sequencing reads. Current practical methods are based on heuristics which are hard to analyse and thus such analysis is not readily available.

**Results:**

We present a model for estimating the probability of misassembly at each position of a de Bruijn graph based assembly. Unlike previous work, our model also takes into account missing data. We apply our model to produce contigs with correctness guarantee and correctness estimates for each position in the contigs.

**Conclusions:**

Our experiments show that when the coverage of *k*-mers is high enough, our method produces contigs with similar contiguity characteristics as state-of-the-art assemblers which are based on heuristic correction of the de Bruijn graph. Our model may have further applications in downstream analysis of contigs or in any analysis working directly on the de Bruijn graph.

## Background

Genome assembly is a classical problem in computational biology where the task is to reconstruct a genome based on sequencing reads. Theoretically, genome assembly has been formulated as finding the shortest common superstring of the reads, finding a Hamiltonian path in a string graph, or finding a Eulerian tour in a de Bruijn graph. However, the solutions to these problems are almost never unique, and thus practical methods are instead based on heuristics. These heuristics are hard to analyse and thus such analysis is not readily available [1]. Furthermore, it has been shown that misassemblies are present in the output sequences due to not considering structural correctness properly [2].

Current solutions produce a set of sequences that are claimed to be substrings of the genome. Most assemblers are based on unitigs which are strings corresponding to nonbranching paths in a string graph or a de Bruijn graph and thus guaranteed to be substrings of the genome if there is no missing data, i.e. missing branches in the graph. A further theoretical development is to produce omnitigs [3] which are guaranteed to be substrings of each genome that could produce the string graph or de Bruijn graph but again it is assumed there is no missing data. In the presence of missing data, these approaches may report strings that are not substrings of the original genome.

Several works have previously investigated under which conditions a genome assembly is correct. It has for example been shown that if enough information is available, a unique and correct solution can be found in polynomial time [4, 5]. Enough information can be characterized as the solution being unique [5] or requiring that all repeat instances of the genome are spanned by some read [4]. However, the requirement for enough information essentially means that there can be no missing data and these approaches do not easily extend to estimating the probability of correctness for a given position in the assembly.

Probabilistic frameworks have previously been applied to genome assembly to produce longer contigs. Medvedev and Brudno [6] and Myers [7] have proposed to report the sequence with maximum likelihood given the reads as the assembled genome and Boža et al. [8] have developed a probabilistic framework to produce an assembly with high likelihood using simulated annealing. However, these approaches do not assign probabilities to misassemblies nor do they consider missing data. Howison et al. [9] have proposed to use a Markov chain Monte Carlo approach to providing posterior probabilities of assemblies but their approach is computationally expensive and thus they only applied it to the bacteriophage $\Phi$X174. Finally, the correctness of genome assembly has been studied in the context of validating the produced assembly [10, 11].

We present a model for structural correctness in de Bruijn graph based assembly. Our model estimates the probability of misassembly for each edge in the de Bruijn graph. We apply our model to de Bruijn graph based assembly to create an assembled genome with a correctness guarantee and a correctness estimate for each position in the assembly. We call the resulting assembler Sequence Assembly avoiding MisAssemblies (SAMA). Although here we apply the model to the genome assembly problem, the model can be of independent interest beyond assembly and could be useful for any downstream analysis based on the results of assembly or to any analysis performed directly on a de Bruijn graph. Our experiments show that when *k*-mer coverage is high enough for computing accurate estimates, our method produces as contiguous assemblies as a state-of-the-art assembler based on heuristic correction of the de Bruijn graph like tip and bulge removal [12]. Our method is also competitive in runtime and memory usage.

## Methods

We will first give an overview of our method for de Bruijn graph based assembly with structural correctness guarantees and then we will explain the related analysis and the implementation details of the method.

### Overview of the method

Given a set of reads, we first construct the de Bruijn graph of the reads. The de Bruijn graph contains a node for each *k*-mer occurring in the reads. An edge is added between two *k*-mers if they overlap by $k-1$ characters and the resulting $k+1$-mer occurs in the reads. For each node we compute the abundance of the *k*-mer which is the number of occurrences of the *k*-mer in the read set. Similarly, we also compute the abundance of each edge.

Assembly in a de Bruijn graph is a set of paths where each path corresponds to a contig. Based on the abundances of nodes and edges, we will estimate for each edge the probability that using that edge in an assembly can result in a misassembly. In other words, we estimate the probability that an edge representing an extension of a *k*-mer to a $k+1$-mer is not a unique extension of the *k*-mer in the original genome. If the extension is not unique, using the edge in an assembly can result in misassembly. Finally, given a threshold for the probability of misassembly, we will produce contigs by finding all maximal paths in the de Bruijn graph such that the estimated misassembly probability of each edge in the paths is below the threshold.

### Definitions and problem formulation

Let us consider a set of reads *R* from a genome *G*. We assume that the reads are sampled at random from the genome. We denote the abundance or frequency of a sequence *S* in the read set *R* by *f*(*S*). When we want to emphasize that the abundance is a constant we use *a* to denote the abundance of a sequence.

A *k*-mer is a sequence of length *k*. The *k*-mer spectrum of a read set is the set of all *k*-mers occurring in the read set. We construct the de Bruijn graph for a read set by enumerating its *k*-mer spectrum. Each *k*-mer occurring in the read set is a node of the de Bruijn graph. There is an edge between two *k*-mers $S[1\ldots k]$ and $T[1\ldots k]$ if they overlap by $k-1$ characters, i.e. $S[2\ldots k] = T[1\ldots k-1]$ and the $k+1$-mer $S[1\ldots k]T[k]=S[1]T[1\ldots k]$ also occurs in the read set. An assembly in a de Bruijn graph consists of a set of paths. Each path $S_1S_2\ldots S_n$ corresponds to a sequence consisting of the first *k*-mer in the path concatenated with the last characters of each of the remaining *k*-mers: $S_1[1\ldots k] S_2[k] S_3[k] \ldots S_n[k]$.

Here we are interested in estimating the probability of missassembly for each edge in the de Bruijn graph. Let *S* be a sequence (a *k*-mer) which occurs as a substring in *R* *a* times, i.e. the abundance of *S* in *R* is *a*. Let us assume that also the sequence *Sc* (a $k+1$-mer), where *c* is a nucleotide, occurs as a substring in *R*. Now we are interested in whether *Sc* is a unique extension of *S* in *G* or not. If *Sc* is not a unique extension of *S* in *G*, we should not utilise the edge *Sc* in a de Bruijn graph assembly since the correctness of the edge depends on the context where *Sc* occurs and information about this context is not available in a de Bruijn graph. Note that *Sc* can be a unique extension even when *S* is a repeat in *G*. A unique extension then means that each repeat copy of *S* in *G* is followed by *c* or in other words the multiplicities of *S* and *Sc* are the same in *G*.

We thus need to estimate the following probability. If a *k*-mer *S* is a repeat and *Sc* is not a unique extension of *S*, what is the probability that *Sc* occurs with abundance at least $\ell$ in the read set. Therefore, we solve the following problem:

**Problem 1 **If *S* is a repeat in *G*, what is the probability that the abundance of *Sc* is larger than $\ell$ in *R* when *S* has at least two extensions in *G*, *Sc* and $Sc'$.

This probability estimate directly gives us an estimate for an edge *Sc* to cause a misassembly.

Below we will split our analysis based on the number of repeat copies of *S* in *G*. We denote the number of repeat copies by $\alpha$ and call a repeat with $\alpha$ occurrences in *G* an $\alpha$-repeat. Previous work exists to compute estimates for the proportion of *k*-mers that are $\alpha$-repeats based on the abundance histogram of *k*-mers. We will utilise the Detox [13] method to compute these estimates.

### Probability of misassembly for repeated sequences

We assume here that all sequences that occur with frequency at least $\ell _e$ in the read set *R* are truly subsequences of the genome *G*. However, some of the subsequences of the genome *G* might not occur in *R*. We now want to analyse what is the probability of *Sc* not being a unique extension of *S* in *G* if the frequency of *S* and *Sc* are above some threshold $\ell \ge \ell _e$ in *R*.

If the sequence *S* is not a repeat in *G*, then the probability that *Sc* is not a unique extension of *S* in *G* is 0 since there is a single occurrence of *S* in *G*. Next we will analyse the probability of an extension *Sc* of *S* not to be unique in *G* when *S* is a repeat and the frequency of *S* and *Sc* in *R* is above some threshold $\ell \ge \ell _e$.

#### $\alpha$-repeats

The analysis given here is an adaptation of the analysis given in [14] for 2-repeats.

Let us assume that *S* is an $\alpha$-repeat and thus *S* occurs in *G* $\alpha$ times. *S* then has $\alpha$ occurrences and $\alpha$ extensions in *G*: $Sc_1, Sc_2, \ldots , Sc_\alpha$. In the worst case $\alpha -1$ of these extensions are equal and one is different, i.e. $c_1\ne c_2=c_3=\ldots =c_\alpha$. Since *S* is an $\alpha$-repeat, we require that its extensions occur $\ell>\frac{\alpha -1}{\alpha } a$ times in the read set, where *a* is the abundance of *S* in the read set. For a lower threshold we would be very likely to ignore at least one extension in *G*. Now the abundance of *Sc*, where $c=c_2=c_3=\ldots =c_\alpha$, is distributed as $\text {Binomial}(a,(\alpha -1)/\alpha )$ and thus the probability that the abundance of *Sc* is at least $\ell$ is

1 $$\begin{aligned} P(f(Sc) \ge \ell ) = \sum _{i=\ell }^a \left( {\begin{array}{c}a\\ i\end{array}}\right) \left( \frac{\alpha -1}{\alpha }\right) ^i \left( \frac{1}{\alpha } \right) ^{a-i} \end{aligned}$$

Using the right-tail upper bound by Ferrante [15], we get

2 $$\begin{aligned} P(f(Sc) \ge \ell ) \le \frac{1}{1-r\left( \frac{\ell }{a}, \frac{\alpha -1}{\alpha }\right) } \frac{1}{\sqrt{2\pi \frac{\ell }{a} \left( 1-\frac{\ell }{a}\right) a}} e^{-aD\left( \frac{\ell }{a} \parallel \frac{\alpha -1}{\alpha }\right) } \end{aligned}$$

where $r\left( \frac{\ell }{a}, \frac{\alpha -1}{\alpha }\right)$ is the odds ratio

3 $$\begin{aligned} r\left( \frac{\ell }{a}, \frac{\alpha -1}{\alpha }\right) = \frac{\frac{\alpha -1}{\alpha } \left( 1-\frac{\ell }{a}\right) }{\frac{\ell }{a} \left( 1-\frac{\alpha -1}{\alpha } \right) } \end{aligned}$$

and $D\left( \frac{\ell }{a} \parallel \frac{\alpha -1}{\alpha }\right)$ is the Kullback–Leibler divergence between two Bernoulli random variables

4 $$\begin{aligned} D\left( \frac{\ell }{a} \parallel \frac{\alpha -1}{\alpha }\right) = \frac{\ell }{a} \log \frac{\frac{\ell }{a}}{\frac{\alpha -1}{\alpha }} + \left( 1-\frac{\ell }{a}\right) \log \frac{1-\frac{\ell }{a}}{a-\frac{\alpha -1}{\alpha }}. \end{aligned}$$

Since there are $\alpha$ possible extensions of *S* in *G* for which the above scenario could hold, then the probability that an extension of *S* has an abundance above $\ell$ in *R* is

5 $$\begin{aligned} \alpha P(f(Sc_1) \ge \ell ) \le \alpha \frac{1}{1-r\left( \frac{\ell }{a}, \frac{\alpha -1}{\alpha }\right) } \frac{1}{\sqrt{2\pi \frac{\ell }{a} \left( 1-\frac{\ell }{a}\right) a}} e^{-aD\left( \frac{\ell }{a} \parallel \frac{\alpha -1}{\alpha }\right) } \end{aligned}$$

#### Putting it all together

Now that we have an estimate for the proportions of $\alpha$-repeats for different $\alpha$ and the estimates for an extension of *S* not to be unique in *G* given that *S* is an $\alpha$-repeat, we can compute the total probability that an extension of *S* is not unique:

$$\begin{aligned} P(f(Sc)\ge \ell ) =&P(\alpha =1) P(f(Sc) \ge \ell \mid \alpha =1) \\&+ \sum _{\alpha '=2}^{\infty } P(\alpha =\alpha ') P(f(Sc) \ge \ell \mid \alpha =\alpha ')\\ \le&\sum _{\alpha '=2}^{\infty } P(\alpha =\alpha ') \alpha ' \frac{1}{1-r(\frac{\ell }{a}, \frac{\alpha '-1}{\alpha '})} \frac{1}{\sqrt{2\pi \frac{\ell }{a} \left( 1-\frac{\ell }{a}\right) a}} e^{-aD\left( \frac{\ell }{a} \parallel \frac{\alpha '-1}{\alpha '}\right) } \\ \end{aligned}$$

### Integrating the analysis in de Bruijn graph based assembler

We will now use the results of the above analysis to create a de Bruijn graph based assembly where the probability of misassembly at each position is bounded by a constant $\varepsilon$. We call the resulting tool Sequence Assembly avoiding MisAssemblies (SAMA).

First we construct the de Bruijn graph using BCALM 2 [16]. We then use Detox [13] to estimate for each abundance level *a* and each $\alpha$ the probability that a *k*-mer appearing *a* times in the reads repeats $\alpha$ times in the genome. In practice, Detox limits the values of $\alpha$ in the range [0, 5]. Now we have all that is needed for the analysis presented above.

Given a *k*-mer *S* that is a node of the de Bruijn graph, each edge originating at that node presents an extension of *S* by one character. The above analysis gives us the probability that such an extension is not a unique extension of *S* in the genome given the abundance *a* of *S* and the abundance $a_e$ of *Sc*. We could compute these probabilities for each edge but instead we will use the following more efficient strategy. We can compute a lower bound for the abundance of an extension given the abundance of a *k*-mer such that if an extension has abundance at least equal to the lower bound, then the probability that the extension is not a unique extension is lower than the misassembly threshold $\varepsilon$. These lower bounds can then be applied to any *k*-mer with the given abundance. Hence the next step is to compute such thresholds for each abundance of a *k*-mer. We thus iterate over all abundances of *k*-mers and for each abundance we find the lowest abundance of an extension for which the probability of an extension not being unique is less than the parameter $\varepsilon$, our probability threshold for misassembly.

**Fig. 1:**
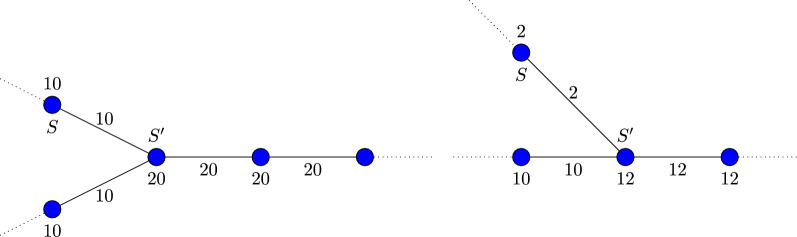
Typical situations where the edge of the de Bruijn graph is a unique extension in one direction but not a unique extension in the reverse direction. In the left figure the node *S* is a unique *k*-mer in the genome but $S'$ is a repeat *k*-mer. Then *S* can be extended towards $S'$ uniquely but $S'$ cannot be extended towards *S* uniquely. Similarly in the right figure the node *S* is an erroneous *k*-mer that does not occur in the genome and $S'$ is a unique *k*-mer in the genome. Now our analysis will denote the extension of *S* towards $S'$ as a unique extension but not the extension of $S'$ towards *S*

The final step is to generate contigs where each de Bruijn graph edge included in the contigs meets the abundance threshold. We note that an edge between two de Bruijn graph nodes *S* and $S'$ can meet the abundance threshold in one direction but not in the other. This commonly happens at the boundary of repeats, where e.g. *S* is not a repeat *k*-mer but $S'$ is (see Fig. 1 left), and at the boundary of sequencing errors, where e.g. *S* is an erroneous *k*-mer but $S'$ is not (see Fig. 1 right). Then we can extend *S* towards $S'$ uniquely but we cannot extend $S'$ uniquely towards *S*. When generating contigs we only utilize edges which are unique extensions in both directions. We further note that the thresholds computed in the previous stage are always larger than half of the abundance of the node and therefore, at most one edge out of the node can meet the abundance threshold. Therefore, if using only such edges there is no branches to resolve.

We use the following procedure to traverse the paths of the de Bruijn graph to generate contigs. We ignore nodes with an abundance lower than five since these are likely to be results of sequencing errors. We start the traversal in some arbitrary node. We first traverse edges to the right from the chosen node as long as we find edges that meet the abundance threshold. Then we do a similar traversal to the left. During the traversal we mark each visited node to avoid producing duplicate contigs. When both traversals are complete, we produce a contig corresponding to the traversed path. We then iterate and start a new traversal from an arbitrary node that has not yet been marked (i.e. is not part of a previously generated contig). The contig generation finishes when all nodes have been marked.

## Experiments

### Datasets and methods

We ran experiments on real HiFi reads from *E. coli* and real Illumina reads from *S. aureus*. Both datasets were downsampled to the desired coverage levels. To investigate the performance of our model and other assemblers on different coverage levels, we produced three datasets of the *E. coli* dataset Ecoli20x, Ecoli40x, and Ecoli80x with coverages 20x, 40x, and 80x, respectively. The *S. aureus* was downsampled to 80x coverage. Additionally, The *E. coli* and *S. aureus* datasets have been downsampled to achieve the desired coverage we simulated two datasets on human chromosome 21 with Art [17]: HumanChr21-40x with 40x coverage and HumanChr21-60x with 60x coverage. The details of the datasets are in Table 1.

**Table 1:** Details of the datasets

Name	Organism	Ref seq	Ref seq	Accession	Technology	Coverage	Total read
			length				length
			(Mb)				(Mb)
Ecoli20x	*E. coli* K12	NC_000913	4.6	SRR10971019	HiFi	20x	92.0
	MG 1655						
Ecoli40x	*E. coli* K12	NC_000913	4.6	SRR10971019	HiFi	40x	184.0
	MG 1655						
Ecoli80x	*E. coli* K12	NC_000913	4.6	SRR10971019	HiFi	80x	371.3
	MG 1655						
Saureus	*S. aureus*	CP026067	2.9	SRR1955629	Illumina	80x	230.5
	NRS153						
HumanChr21-40x	*H. sapiens*	CP068257	45.1	Simulated	Illumina	40x	1,803.6
	CHM13 chr 21						
HumanChr21-60x	*H. sapiens*	CP068257	45.1	Simulated	Illumina	60x	2,705.4
	CHM13 chr 21						

The human chromosome 21 datasets are simulated with ART [17]. The reference sequence lengths and total read lengths are given in millions of bases (Mb). *E. coli S. aureus* datasets have been downsampled to achieve the desired coverage

We compared SAMA to unitigs produced by BCALM 2 [16] and contigs produced by SPAdes [18] without error correction and before repeat resolution (which reuses the reads by mapping them on the de Bruijn graph using then the full read information to resolve repeats). BCALM 2 thus represents traditional unitigs and SPAdes contigs results of an assembly algorithm applying tip and bulge removal.

We ran all methods with two values of *k*, 31 and 63. We chose odd values because SPAdes and SAMA require *k* to be odd. Furthermore, 31-mers and 63-mers allow efficient representation of *k*-mers as 64-bit and 128-bit integers, respectively. Experiments shown in Supplementary Table 1 show that the continuity of assembly produced by SAMA gradually increases when *k* is increased. SAMA was run with six values for the misassembly threshold $\varepsilon$: 0.4, 0.2, 0.1, 0.01, 0.001, and 0.0001. BCALM 2 was run with three abundance thresholds: 5, 10, and 15. SPAdes was run with the –only-assembler option to disable error correction and the contigs saved in the before_rr.fasta file were analysed. We used QUAST [19] to produce statistics on the produced assemblies. All experiments were run on a computer with 32 GB of memory and 4 CPUs each with 2 cores.

### Abundance thresholds

**Fig. 2:**
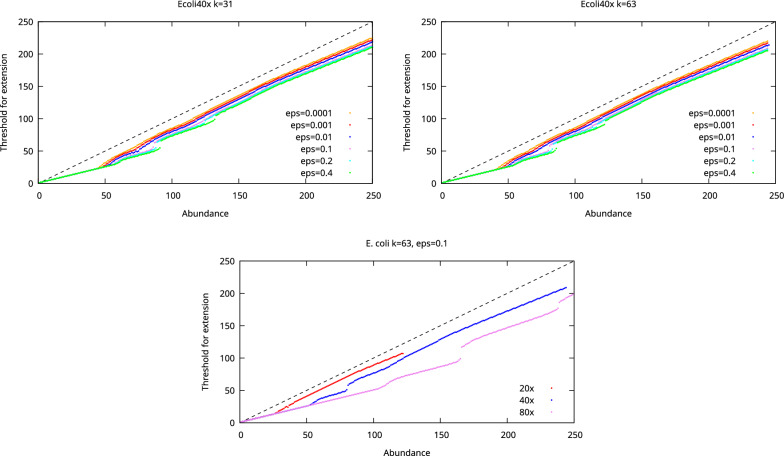
The threshold for extension as a function of *k*-mer abundance when the allowed probability for misassembly is varied (top) and when the coverage of the dataset is varied (bottom) in the *E. coli* datasets. The dashed line shows the maximum possible threshold. Missing dots indicate *k*-mer abundances where no threshold guaranteeing the desired misassembly probability can be found

**Fig. 3:**
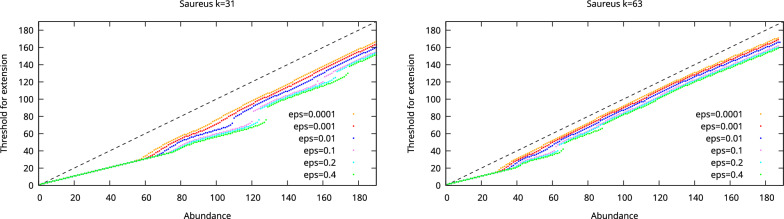
The threshold for extension as a function of *k*-mer abundance when the allowed probability for misassembly is varied for the Saureus dataset. The dashed line shows the maximum possible threshold. Missing dots indicate *k*-mer abundances where no threshold guaranteeing the desired misassembly probability can be found

**Fig. 4:**
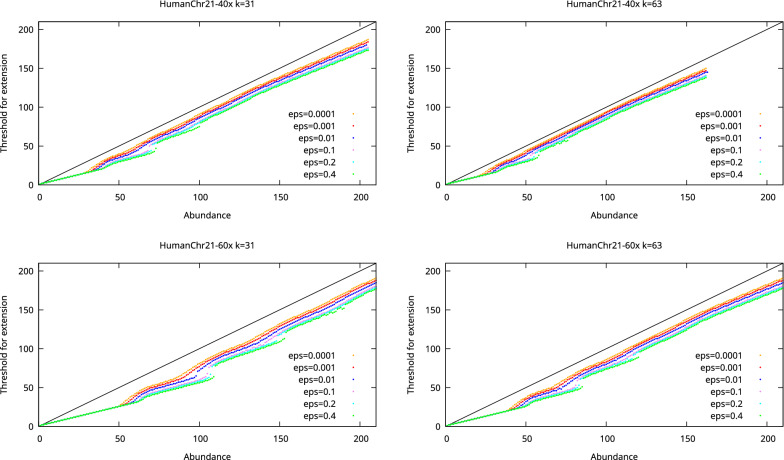
The threshold for extension as a function of *k*-mer abundance when the allowed probability for misassembly is varied for the HumanChr21-40x dataset (top) and Human Chr21-60x dataset (bottom). The dashed line shows the maximum possible threshold. Missing dots indicate *k*-mer abundances where no threshold guaranteeing the desired misassembly probability can be found

We first examined the abundance thresholds produced by our analysis. The thresholds as a function of *k*-mer abundance for the *E. coli*, *S. aureus*, and human chromosome 21 datasets are shown in Figs. 2, 3, and 4, respectively. These thresholds thus indicate what is the minimum abundance of a $k+1$-mer for it to be considered as a unique extension of a *k*-mer.

For low abundance *k*-mers the thresholds are close to half the abundance of the *k*-mers because these *k*-mers are likely to be unique in the genome and for the unique *k*-mers probability of misassembly in our model is 0. For the higher abundance *k*-mers, the thresholds are close to the abundance of the *k*-mers due to most of these *k*-mers originating from repeats and then a lot of evidence is needed to determine that the extension is likely to be unique. However, the threshold does not increase evenly which is due to the proportions of repeats with different number of occurrences changing when the abundance of *k*-mers increases. We also see that the thresholds increase when $\varepsilon$ increases. When the probability of misassembly is set low, we find some *k*-mer abundances for which no abundancy threshold exist such that we could guarantee a low enough misassembly probability.

### Comparison to other methods

We computed the following statistics on all the assemblies produced by SAMA, BCALM 2, and SPAdes:

- *#contigs:* The number of contigs longer than 100 bp in the assembly.
- *NGA50:* The contigs are first aligned on the reference genome. The alignments are then ordered from largest to smallest. The NGA50 metric is the length of the alignment where the cumulative length of the alignments exceeds half the genome length.
- *Misassemblies:* The number of misassemblies as reported by QUAST.
- *Genome fraction:* The percentage of nucleotides in the genome that are covered by at least one contig.

Table 2 shows the statistics of the assemblies produced by the various methods on the *E. coli* datasets. Similarly, Table 3 shows the statistics of the assemblies produced by the methods on the Saureus and human chromosome 21 datasets.

We see that SAMA produces more contiguous assemblies, i.e. lower number of contigs, higher NGA50, and higher genome fraction, when we allow a higher probability for misassemblies. SAMA produces misassemblies only on the HumanChr21-40x and HumanChr21-60x datasets when the allowed probability for misassemblies is high. When the allowed probability of misassemblies is less than or equal to 0.01 there are no misassemblies on any of the datasets.

SAMA produces more contiguous assemblies than the unitigs produced by BCALM 2 when the allowed probability for misassemblies is set appropriately. When the coverage of the dataset is high enough for accurate enough probability estimates, SAMA produces assemblies with similar contiguity characteristics as SPAdes and with no misassemblies. When coverage is lower (Ecoli20x and Saureus datasets), SPAdes produces somewhat more contiguous assemblies. On the other hand, on the more complex human chromosome 21 datasets, SAMA produces more contiguous assemblies than SPAdes. It is worth noting that although the Saureus dataset has nucleotide coverage of 80x, the read length is only 101 bp, and so the coverage of *k*-mers is only 56x and 31x when *k* is 31 and 63, respectively.

**Table 2:** Assembly statistics for the *E. coli* datasets

Dataset	Method	Parameters	#contigs	NGA50	Misassemblies	Genome
			($\ge$ 100 bp)			fraction (%)
Ecoli20x	SAMA	k=31 $\varepsilon$=0.4	464	22,173	0	97.5
	SAMA	k=31 $\varepsilon$=0.2	485	21,266	0	97.5
	SAMA	k=31 $\varepsilon$=0.1	499	20,037	0	97.5
	SAMA	k=31 $\varepsilon$=0.01	515	20,037	0	97.5
	SAMA	k=31 $\varepsilon$=0.001	531	19,183	0	97.5
	SAMA	k=31 $\varepsilon$=0.0001	569	16,972	0	97.5
	SAMA	k=63 $\varepsilon$=0.4	538	60,614	0	98.5
	SAMA	k=63 $\varepsilon$=0.2	548	60,614	0	98.5
	SAMA	k=63 $\varepsilon$=0.1	555	60,614	0	98.5
	SAMA	k=63 $\varepsilon$=0.01	572	59,680	0	98.5
	SAMA	k=63 $\varepsilon$=0.001	588	57,852	0	98.5
	SAMA	k=63 $\varepsilon$=0.0001	648	45,813	0	98.5
	BCALM2	k=31 a=5	747	13,532	0	97.6
	BCALM2	k=31 a=10	1,101	15,302	0	96.3
	BCALM2	k=31 a=15	4,188	2,413	0	78.4
	BCALM2	k=63 a=5	874	31,596	0	98.7
	BCALM2	k=63 a=10	1,660	26,917	0	97.1
	BCALM2	k=63 a=15	6,231	950	0	77.2
	SPAdes	k=31	469	22,175	0	97.1
	SPAdes	k=63	259	78,620	0	98.2
Ecoli40x	SAMA	k=31 $\varepsilon$=0.4	469	23,326	0	97.6
	SAMA	k=31 $\varepsilon$=0.2	474	22,902	0	97.6
	SAMA	k=31 $\varepsilon$=0.1	476	22,902	0	97.6
	SAMA	k=31 $\varepsilon$=0.01	501	22,173	0	97.6
	SAMA	k=31 $\varepsilon$=0.001	507	22,173	0	97.6
	SAMA	k=31 $\varepsilon$=0.0001	510	21,793	0	97.6
	SAMA	k=63 $\varepsilon$=0.4	1,385	78,618	0	98.7
	SAMA	k=63 $\varepsilon$=0.2	1,391	78,618	0	98.7
	SAMA	k=63 $\varepsilon$=0.1	1,397	78,618	0	98.8
	SAMA	k=63 $\varepsilon$=0.01	1,410	73,700	0	98.8
	SAMA	k=63 $\varepsilon$=0.001	1,432	61,690	0	98.8
	SAMA	k=63 $\varepsilon$=0.0001	1,443	57,852	0	98.8
	BCALM2	k=31 a=5	2112	4,082	0	97.5
	BCALM2	k=31 a=10	576	18,227	0	97.6
	BCALM2	k=31 a=15	541	21,235	0	97.7
	BCALM2	k=63 a=5	3751	5,702	0	98.9
	BCALM2	k=63 a=10	583	59,552	0	98.7
	BCALM2	k=63 a=15	542	63,602	0	98.7
	SPAdes	k=31	485	22,175	0	97.8
	SPAdes	k=63	274	78,620	0	98.4

Dataset	Method	Parameters	#contigs	NGA50	Misassemblies	Genome
			($\ge$ 100 bp)			fraction (%)
Ecoli80x	SAMA	k=31 $\varepsilon$=0.4	488	23,334	0	97.7
	SAMA	k=31 $\varepsilon$=0.2	501	23,326	0	97.7
	SAMA	k=31 $\varepsilon$=0.1	512	22,902	0	97.7
	SAMA	k=31 $\varepsilon$=0.01	513	22,902	0	97.6
	SAMA	k=31 $\varepsilon$=0.001	514	22,902	0	97.6
	SAMA	k=31 $\varepsilon$=0.0001	513	22,902	0	97.6
	SAMA	k=63 $\varepsilon$=0.4	6,664	78,618	0	99.1
	SAMA	k=63 $\varepsilon$=0.2	6,675	78,618	0	99.1
	SAMA	k=63 $\varepsilon$=0.1	6,691	78,618	0	99.1
	SAMA	k=63 $\varepsilon$=0.01	6,712	78,618	0	99.1
	SAMA	k=63 $\varepsilon$=0.001	6,716	78,618	0	99.1
	SAMA	k=63 $\varepsilon$=0.0001	6,719	78,618	0	99.1
	BCALM2	k=31 a=5	8,019	813	0	95.4
	BCALM2	k=31 a=10	1,465	6,189	0	97.5
	BCALM2	k=31 a=15	653	16,190	0	97.6
	BCALM2	k=63 a=5	19,389	986	0	99.0
	BCALM2	k=63 a=10	2,275	9,466	0	98.8
	BCALM2	k=63 a=15	723	41,980	0	98.8
	SPAdes	k=31	485	22,175	0	97.8
	SPAdes	k=63	268	78,620	0	98.3

**Table 3:** Assembly statistics for the Saureus and HumanChr21 datasets

Dataset	Method	Parameters	#contigs	NGA50	Misassemblies	Genome
			($\ge$ 100 bp)			fraction (%)
Saureus	SAMA	k=31 $\varepsilon$=0.4	392	24,807	0	96.9
	SAMA	k=31 $\varepsilon$=0.2	392	23,869	0	96.9
	SAMA	k=31 $\varepsilon$=0.1	394	23,768	0	96.9
	SAMA	k=31 $\varepsilon$=0.01	391	23,768	0	96.8
	SAMA	k=31 $\varepsilon$=0.001	390	23,768	0	96.8
	SAMA	k=31 $\varepsilon$=0.0001	395	21,519	0	96.8
	SAMA	k=63 $\varepsilon$=0.4	427	31,697	0	98.1
	SAMA	k=63 $\varepsilon$=0.2	432	31,697	0	98.1
	SAMA	k=63 $\varepsilon$=0.1	435	31,697	0	98.1
	SAMA	k=63 $\varepsilon$=0.01	446	31,697	0	98.1
	SAMA	k=63 $\varepsilon$=0.001	459	31,260	0	98.1
	SAMA	k=63 $\varepsilon$=0.0001	485	31,028	0	98.1
	BCALM2	k=31 a=5	687	9,337	0	96.7
	BCALM2	k=31 a=10	484	18,684	0	96.8
	BCALM2	k=31 a=15	422	22-009	0	96.9
	BCALM2	k=63 a=5	643	17,374	0	98.2
	BCALM2	k=63 a=10	906	8,882	0	98.2
	BCALM2	k=63 a=15	4,965	1,350	0	97.8
	SPAdes	k=31	453	26,628	0	97.1
	SPAdes	k=63	264	39,224	0	97.9
HumanChr21-40x	SAMA	k=31 $\varepsilon$=0.4	44,175	1,446	0	74.6
	SAMA	k=31 $\varepsilon$=0.2	43,877	1,439	0	74.3
	SAMA	k=31 $\varepsilon$=0.1	43,750	1,434	0	74.2
	SAMA	k=31 $\varepsilon$=0.01	43,612	1,430	0	74.2
	SAMA	k=31 $\varepsilon$=0.001	43,635	1,426	0	74.1
	SAMA	k=31 $\varepsilon$=0.0001	43,664	1,423	0	74.1
	SAMA	k=63 $\varepsilon$=0.4	37,689	8,336	1	90.0
	SAMA	k=63 $\varepsilon$=0.2	38,313	8,268	1	89.9
	SAMA	k=63 $\varepsilon$=0.1	38,681	8,201	1	89.9
	SAMA	k=63 $\varepsilon$=0.01	38,979	8,158	0	89.9
	SAMA	k=63 $\varepsilon$=0.001	39,040	8,151	0	89.9
	SAMA	k=63 $\varepsilon$=0.0001	39,083	8,131	0	89.9
	BCALM2	k=31 a=5	43,796	1,351	0	73.4
	BCALM2	k=31 a=10	44,041	1,352	0	73.5
	BCALM2	k=31 a=15	45,753	1,220	0	73.4
	BCALM2	k=63 a=5	40,051	7,687	0	89.9
	BCALM2	k=63 a=10	41,471	5,945	0	90.0
	BCALM2	k=63 a=15	72,222	1,166	0	89.0
	SPAdes	k=31	38,604	1,455	0	74.1
	SPAdes	k=63	23,125	8,187	0	87.1
HumanChr21-60x	SAMA	k=31 $\varepsilon$=0.4	44,123	1,448	1	74.6
	SAMA	k=31 $\varepsilon$=0.2	43,868	1,440	0	74.4
	SAMA	k=31 $\varepsilon$=0.1	43,715	1,436	0	74.2
	SAMA	k=31 $\varepsilon$=0.01	43,603	1,431	0	74.2
	SAMA	k=31 $\varepsilon$=0.001	43,597	1,430	0	74.1
	SAMA	k=31 $\varepsilon$=0.0001	43,596	1,429	0	74.1
	SAMA	k=63 $\varepsilon$=0.4	39,586	8,300	1	90.3
	SAMA	k=63 $\varepsilon$=0.2	40,232	8,230	0	90.2
	SAMA	k=63 $\varepsilon$=0.1	40,526	8,194	0	90.2
	SAMA	k=63 $\varepsilon$=0.01	40,833	8,166	0	90.2
	SAMA	k=63 $\varepsilon$=0.001	40,856	8,164	0	90.2
	SAMA	k=63 $\varepsilon$=0.0001	40,862	8,164	0	90.2
	BCALM2	k=31 a=5	43,691	1,347	0	73.3
	BCALM2	k=31 a=10	43,974	1,352	0	73.5
	BCALM2	k=31 a=15	44,015	1,354	0	73.5
	BCALM2	k=63 a=5	41,037	7,693	0	90.0
	BCALM2	k=63 a=10	40,025	7,693	0	90.0
	BCALM2	k=63 a=15	40,034	7,509	0	90.0
	SPAdes	k=31	38,404	1,455	0	74.1
	SPAdes	k=63	22,990	8,187	0	87.1

Table 4 shows the runtime and peak memory usage of the tools. The statistics for SAMA include running BCALM 2 and Detox since they are part of the needed pipeline. The comparison against SPAdes is not entirely fair because it was not possible to disable running the repeat resolution code which uses a significant amount of resources. We see that SAMA is competitive in both runtime and memory usage.

**Table 4:** Runtime and memory usage

Dataset	Method	Parameters	Runtime (min)	Memory Usage (GB)
Ecoli20x	SAMA	k=31 $\varepsilon$=1e-01	0.89	1.28
	SAMA	k=63 $\varepsilon$=1e-01	0.87	1.35
	BCALM2	k=31 a=10	0.23	0.58
	BCALM2	k=63 a=5	0.32	0.87
	SPAdes	k=31	0.50	0.79
	SPAdes	k=63	0.84	1.53
Ecoli40x	SAMA	k=31 $\varepsilon$=1e-01	1.17	1.46
	SAMA	k=63 $\varepsilon$=1e-01	1.33	1.67
	BCALM2	k=31 a=15	0.35	0.98
	BCALM2	k=63 a=15	0.44	1.55
	SPAdes	k=31	0.97	1.54
	SPAdes	k=63	1.64	2.21
Ecoli80x	SAMA	k=31 $\varepsilon$=1e-01	1.92	1.94
	SAMA	k=63 $\varepsilon$=1e-01	2.26	2.88
	BCALM2	k=31 a=15	0.54	1.71
	BCALM2	k=63 a=15	0.68	2.88
	SPAdes	k=31	1.83	2.21
	SPAdes	k=63	3.29	2.22
Saureus	SAMA	k=31 $\varepsilon$=1e-01	0.95	0.88
	SAMA	k=63 $\varepsilon$=1e-01	0.75	0.81
	BCALM2	k=31 a=15	0.28	0.76
	BCALM2	k=63 a=5	0.28	0.76
	SPAdes	k=31	0.90	1.34
	SPAdes	k=63	0.66	1.45
HumanChr21-40x	SAMA	k=31 $\varepsilon$=1e-01	20.76	11.49
	SAMA	k=63 $\varepsilon$=1e-01	1307	11.78
	BCALM2	k=31 a=10	2.86	3.23
	BCALM2	k=63 a=5	3.32	3.34
	SPAdes	k=31	132.93	18.79
	SPAdes	k=63	32.77	6.92
HumanChr21-60x	SAMA	k=31 $\varepsilon$=1e-01	24.68	12.79
	SAMA	k=63 $\varepsilon$=1e-01	19.34	12.91
	BCALM2	k=31 a=15	3.78	4.17
	BCALM2	k=63 a=5	4.28	4.30
	SPAdes	k=31	167.78	25.62
	SPAdes	k=63	47.21	9.53

## Conclusion

We have presented a model to estimate the probability of misassembly at each position of a de Bruijn graph based assembly. As far as we know, this is the first model that can assign such a probability to each position in the assembly also taking into account missing data. We applied our model to produce contigs with correctness guarantee and showed that this leads to a practical method. Here we applied the model to produce an assembly where the probability of misassembly at each position is bounded. However, this information has further potential applications in the downstream analysis of a genome and in any analysis method working directly on the de Bruijn graph. For example, the probabilities computed according to our model could be used to evaluate the confidence of structural variants or genome rearrangements detected in an assembly. Many analysis methods working directly on a de Bruijn graph align sequences to the graph. The probabilities computed according to our model could be used to assign confidence values to these alignments and possibly favor alignments that span edges with high probabilities of correctness.

Our current model is limited to single haploid genomes. It would be interesting to extend the ideas presented here to diploid genomes as well as viral and bacterial metagenomics.

## Supplementary Information

Supplementary Material 1.

## Data Availability

The datasets for the experiments were obtained from public repositories. See the corresponding accession numbers in Table 1.

## Notes

The software accompanying this article is available in GitHub: • Project name: SAMA. • Project home page: https://github.com/LeenaSalmela/SAMA. • Operating system(s): Linux. • Programming language: C++. • Other requirements: CMake version 2.6.3 or higher, GCC version 4.7 or higher, Google’s sparsehash, ZLIB (optional), recent Boost libraries (for approximate inference), GMP library (for approximate inference) •. License: GNU AGPL.

## References

[1] Medvedev P. Theoretical analysis of sequencing bioinformatics algorithms and beyond. Commun ACM. 2023;66(7):118–25. 38736702 10.1145/3571723 PMC11087067

[2] Rahman A, Medvedev P. Assembler artifacts include misassembly because of unsafe unitigs and under-assembly because of bidirected graphs. Genome Res. 2022;32:1746–53. 35896425 10.1101/gr.276601.122 PMC9528984

[3] Tomescu AI, Medvedev P. Safe and complete contig assembly via omnitigs. In: Singh M, editor. Research in Computational Molecular Biology. Cham: Springer; 2016. p. 152–63. 10.1089/cmb.2016.0141 27749096

[4] Nagarajan N, Pop M. Parametric complexity of sequence assembly: theory and applications to next generation sequencing. J Comput Biol. 2009;16(7):897–908. 19580519 10.1089/cmb.2009.0005

[5] Shomorony I, Kim SH, Courtade TA, Tse DNC. Information-optimal genome assembly via sparse read-overlap graphs. Bioinformatics. 2016;32(17):494–502. 10.1093/bioinformatics/btw450. 10.1093/bioinformatics/btw450 27587667

[6] Medvedev P, Brudno M. Maximum likelihood genome assembly. J Comput Biol. 2009;16(8):1101–16. 19645596 10.1089/cmb.2009.0047 PMC3154397

[7] Myers EW. The fragment assembly string graph. Bioinformatics. 2005;21(2):79–85. 10.1093/bioinformatics/bti1114. 10.1093/bioinformatics/bti1114 16204131

[8] Boža V, Brejová B, Vinař T. GAML: genome assembly by maximum likelihood. Algorithms Mol Biol. 2015. 10.1186/s13015-015-0052-6. 26042154 10.1186/s13015-015-0052-6 PMC4454275

[9] Howison M, Zapata F, Edwards EJ, Dunn CW. Bayesian genome assembly and assessment by Markov chain Monte Carlo sampling. PLoS ONE. 2014;9(6):99497. 10.1371/journal.pone.0099497. 10.1371/journal.pone.0099497 PMC4072599 24968249

[10] Rahman A, Pachter L. CGAL: computing genome assembly likelihoods. Genom Biol. 2013;14(R8):1–10. 10.1186/gb-2013-14-1-r8 PMC3663106 23360652

[11] Kim S, Liao L, Tomb J-F, A probabilistic approach to sequence assembly validation. In: Proceedings of BIOKDD, 2001; pp. 38–43

[12] Zerbino DR, Birney E. Velvet: algorithms for de novo short read assembly using de Bruijn graphs. Genome Res. 2008;18:821–9. 18349386 10.1101/gr.074492.107 PMC2336801

[13] Steyaert A, Audenaert P, Fostier J. Improved node and arc multiplicity estimation in de Bruijn graphs using approximate inference in conditional random fields. IEEE/ACM Trans Comput Biol Bioinf. 2023;20(3):1995–2006. 10.1109/TCBB.2022.3229085 37015543

[14] Díaz-Domínguez D, Onodera T, Puglisi SJ, Salmela L, Genome assembly with variable order de Bruijn graphs. bioRxiv 2022 10.1101/2022.09.06.506758https://arxiv.org/abs/https://www.biorxiv.org/content/early/2022/09/07/2022.09.06.506758.full.pdf

[15] Ferrante GC. Bounds on binomial tails with applications. IEEE Trans Inf Theory. 2021;67(12):8273–9. 10.1109/TIT.2021.3115044.

[16] Chikhi R, Limasset A, Medvedev P. Compacting de Bruijn graphs from sequencing data quickly and in low memory. Bioinformatics. 2016;32(12):201–8. 10.1093/bioinformatics/btw279 (https://arxiv.org/abs/https://academic.oup.com/bioinformatics/article-pdf/32/12/i201/49021929/bioinformatics_32_12_i201.pdf). 10.1093/bioinformatics/btw279 PMC4908363 27307618

[17] Huang W, Li L, Myers JR, Marth GT. ART: a next-generation sequencing read simulator. Bioinformatics. 2011;28(4):593–4. 10.1093/bioinformatics/btr708 (https://arxiv.org/abs/https://academic.oup.com/bioinformatics/article-pdf/28/4/593/48879907/bioinformatics_28_4_593.pdf). 22199392 10.1093/bioinformatics/btr708 PMC3278762

[18] Prjibelski A, Antipov D, Meleshko D, Lapidus A, Korobeynikov A. Using spades de novo assembler. Curr Protoc Bioinform. 2020;70(1):102. 10.1002/cpbi.102 (https://arxiv.org/abs/https://currentprotocols.onlinelibrary.wiley.com/doi/pdf/10.1002/cpbi.102). 10.1002/cpbi.102 32559359

[19] Mikheenko A, Prjibelski A, Saveliev V, Antipov D, Gurevich A. Versatile genome assembly evaluation with QUAST-LG. Bioinformatics. 2018;34(13):142–50. 10.1093/bioinformatics/bty266 (https://arxiv.org/abs/https://academic.oup.com/bioinformatics/article-pdf/34/13/i142/50315697/bioinformatics_34_13_i142.pdf). 10.1093/bioinformatics/bty266 PMC6022658 29949969

