# Supplementary material for: Sama: a contig assembler with correctness guarantee
Source: Algorithms Mol Biol. 2025 Jun 3;20:9. doi: 10.1186/s13015-025-00280-y (PMC12135590; doi:10.1186/s13015-025-00280-y)
Supplement: Supplementary file 1 — Supplementary Material 1. [file 13015_2025_280_MOESM1_ESM.pdf]

SAMA: a contig assembler with correctness guarantee Supplementary material

Leena Salmela

Table 1: SAMA assembly statistics for the Ecoli80x dataset with varying values of k. We used $\epsilon = 0.01$ for all these runs. The increase of memory usage from $k = 31$ to $k = 35$ is due to BCALM2 needing more than 64-bits to represent a k-mer.

k	#contigs ($\geq$ 100 bp)	NGA50	Misassemblies	Genome fraction (%)	Runtime (min)	Memory (GB)
31	513	22,902	0	97.6	1.92	1.94
35	484	25,783	0	97.8	2.04	3.02
39	461	32,400	0	97.9	2.08	2.98
43	409	45,447	0	98.0	2.11	2.95
47	391	57,820	0	98.2	2.13	2.92
51	534	60,328	0	98.5	2.17	2.92
55	7,099	60,766	0	99.0	2.19	2.91
59	6,918	63,594	0	99.0	2.25	2.89
63	6,712	78,618	0	99.1	2.26	2.88
